# ON01210.Na (Ex-RAD^®^) Mitigates Radiation Damage through Activation of the AKT Pathway

**DOI:** 10.1371/journal.pone.0058355

**Published:** 2013-03-07

**Authors:** Anthony D. Kang, Stephen C. Cosenza, Marie Bonagura, Manoj Manair, M. V. Ramana Reddy, E. Premkumar Reddy

**Affiliations:** 1 Fels Institute for Cancer Research and Molecular Biology, Philadelphia, Pennsylvania, United States of America; 2 Onconova Therapeutics, Inc., Newtown, Pennsylvania, United States of America; Mizoram University, India

## Abstract

Development of radio-protective agents that are non-toxic is critical in light of ever increasing threats associated with proliferation of nuclear materials, terrorism and occupational risks associated with medical and space exploration. In this communication, we describe the discovery, characterization and mechanism of action of ON01210.Na, which effectively protects mouse and human bone marrow cells from radiation-induced damage both *in vitro* and *in vivo*. Our results show that treatment of normal fibroblasts with ON01210.Na before and after exposure to ionizing radiation provides dose dependent protection against radiation-induced damage. Treatment of mice with ON01210.Na prior to radiation exposure was found to result in a more rapid recovery of their hematopoietic system. The mechanistic studies described here show that ON01210.Na manifests its protective effects through the up-regulation of PI3-Kinase/AKT pathways in cells exposed to radiation. These results suggest that ON 01210.Na is a safe and effective radioprotectant and could be a novel agent for use in radiobiological disasters.

## Introduction

Protection from exposure to ionizing radiation (IR) has become an important issue due to ever increasing threats associated with proliferation of nuclear materials, terrorism and occupational risks associated with medical and space exploration [1]–[2]. The toxic effects of IR can be initially seen in tissues with a high rate of cell division, such as the hematopoietic and gastrointestinal systems, and whole body exposure to IR causes severe acute radiation syndrome in humans [3]. The number of drugs that can protect cells from the effects of IR are limited in number. Amifostine, a drug used as an adjuvant in chemotherapy, is a prodrug that can only be administered intravenously after reconstitution with normal saline and is the only radioprotective agent approved by the FDA. There has been an increase in the efforts to develop potential radiation protectants and mitigators, including novel free radical scavengers, cytokine inhibitors, inhibitors of p53 and other cellular kinases, inhibitors of cell death signaling and modulators of cellular DNA repair mechanism among others [4] .

Due to the limited availability of radioprotective agents, we performed a screen of our small molecule chemical library [5] for compounds that protect cells from the damaging effects of IR using a high throughput cell based assay. From this screen, we have identified several compounds that effectively show radioprotective activity [6] and one of them, ON01210 (4-carboxystyrl-4-chlorbenzysulfone), was found to have the most desirable characteristics for drug development. This novel compound was renamed, Ex-RAD^®^, and has been shown to effectively protect human fibroblast cells (HFL-1) after exposure to IR [6]. The same compound has been tested in mice to show a significant increase in survival rate when the compound is injected before exposure to gamma radiation [7].

In this communication, we describe the discovery, characterization and mechanism of action of ON01210, which effectively protects mouse and human bone marrow cells from radiation-induced damage both *in vitro* and *in vivo*. To gain an understanding of the mechanism of action of this compound, we have examined the signaling pathways associated with radiation-induced damage in the presence and absence of ON01210. Our results demonstrate that ON01210 manifests its protective effects through the up-regulation of PI3-Kinase/AKT pathways in cells exposed to radiation.

## Results

### Compound Screening and *in vitro* Activity

**Figure 1:**
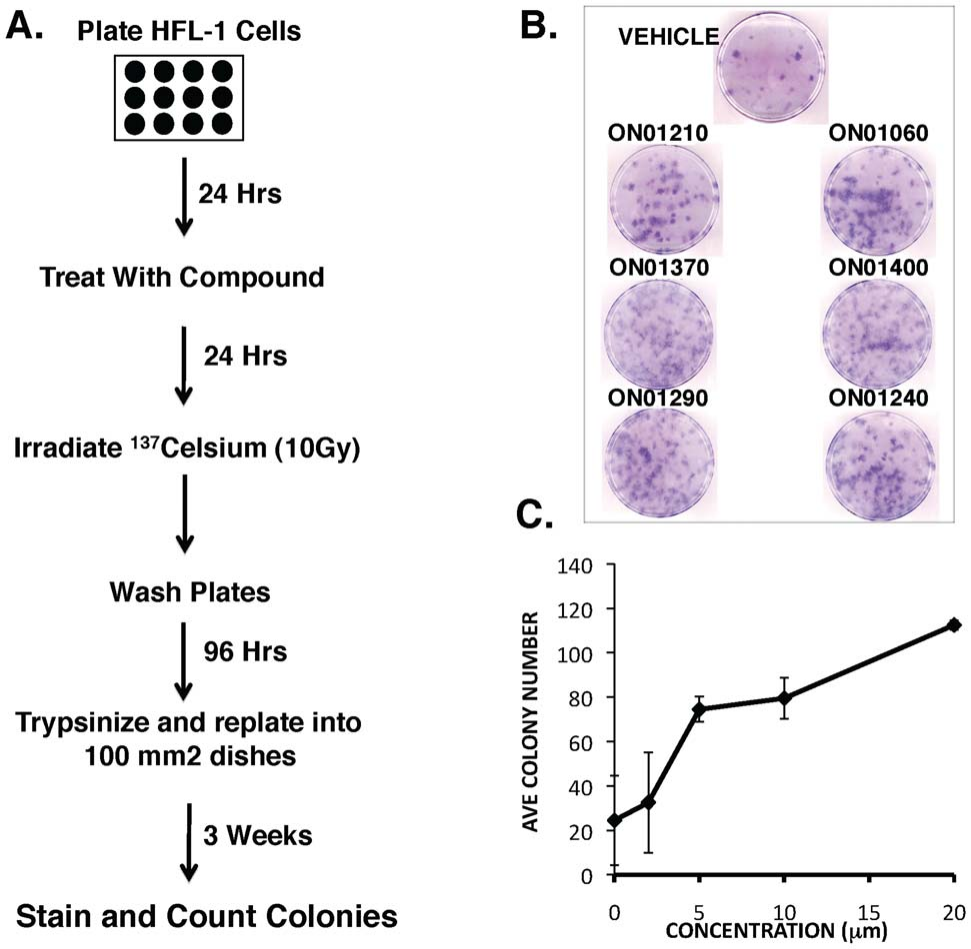
Identification of ON01210 (Ex-RAD^®^) as a radioprotectant from an (E)-Styryl Benzylsulfone chemical library. (A) Screening protocol used to identify radioprotective compounds from the chemical library. HFL-1 cells were pretreated with varying concentrations of compounds for 24 hours before irradiation (10 Gy), followed by replating into fresh dishes to form colonies. (B) Representative results of cells treated with compounds that show radioprotective activity. (C) ON01210 pretreatment protected HFL-1 cells in a dose responsive manner.

To identify compounds that protect cells from the damaging effects of IR, we developed a cell-based screening assay in which normal diploid human fetal lung fibroblasts (HFL-1 cells) were treated for 24 hrs with various concentrations of compounds derived from a focused compound library developed in our laboratory to modulate the activity of mammalian kinases. The cells were then irradiated with 10 Gy of IR which reduces colony forming activity of HFL-1 cells by 90%. The cells were then re-seeded and resulting colonies were counted 3 weeks post-plating (Figure 1A). Using this screening assay, we were able to identify a number of compounds that provided significant protection when compared to vehicle control-treated cells (Figure 1B, Table 1). Of the compounds showing radioprotective activity, ON01210 (4-carboxystyrl-4-chlorbenzysulfone) was selected as the lead compound. Although several other compounds from this library exhibited enhanced radioprotective properties, subsequent pharmacological studies showed that several of these compounds were either poorly bioavailable or exhibited undesirable levels of toxicity. On the other hand, ON01210 was found to be water-soluble as a sodium salt (ON01210.Na), exhibited little or no toxicity in animal models (data not shown) [7]. Importantly, pretreatment of HFL-1 cells with ON01210 resulted radioprotection as evidenced by an in a dose-dependent increase in colony numbers (Figure 1C).

**Table 1:** Protective properties of compounds.

Compound Number	ON Number	Chemical Formula	Fold Protection	GI 50 (uM)
1	ON01060	(E)-4-FLuorostyryl-4-chlorobenzylsulfone	2	2
2	ON01370	(E)-2,4,6-Trimethoxystyryll-4-methoxybenzylsulfone	2.6	0.01
3	ON01410	(E)-2-Methoxystyryl-4-nitrobenzylsulfone	0	ND
4	ON01420	(E)-2,3,4,5,6-Pentafluorostyryl-4-methoxybenzylsulfone	0	ND
5	ON01400	(E)-4-FLuorostyryl-4-trifluoromethylbenzylsulfone	4.5	2.5
6	ON01240	(E)-4-FLuorostyryl-4-cyanobenzylsulfone	4	10
7	ON02010	(Z)-4-FLuorostyryl-4-chlorobenzylsulfone	2	50
8	ON01290	(E)-4-Furanethenyl-2,4-dichlorobenzylsulfone	4	5
9	ON01430	(E)-4-Pyridylethenyl-4-chlorobenzylsulfone	0	ND
10	ON015010	(E)-4-FLuorstyryl-4-chlorophenylsulfone	3	5
11	ON03020	(Z)-Styryl-(E)-2-methoxy-4ethoxystyrylsulfone	2	50
12	ON01200	(E)-4-Hydroxystyryl-4-chlorobenzylsulfone	0	ND
13	ON01210	(E)-4-Carboxystyryl-4-chlorobenzylsulfone	2.6	100

### Cell Cycle and Growth Analysis of Cells Treated with ON01210.Na

**Figure 2:**
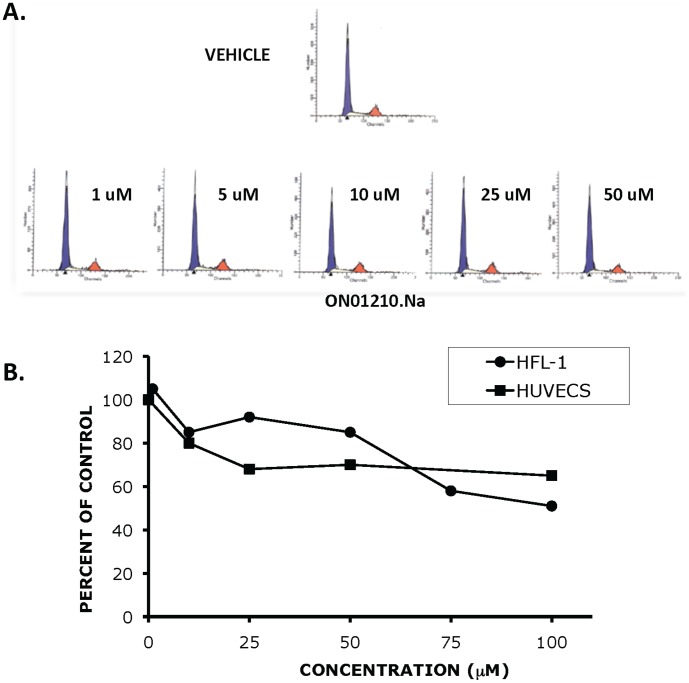
ON01210 Treatment does not alter the cell cycle progression and growth of normal cells (A) HFL-1 cells were treated with increasing concentrations of ON01210 (vehicle alone, 1 uM, 5 uM, 10 uM, 25 uM, and 50 uM) and then subjected to flow cytometric analysis. (B) HFL-1 and human umbilical vein endothelial cells (HUVECS) were plated in the presence of increasing concentrations of ON01210 for 96 hours. The total number of viable cells was determined and the average percent of vehicle control was as a function of concentration.

Because IR is known to cause DNA damage [8], one explanation for the observed radioprotection is that ON01210 places the cells into a “non-sensitive” or pre-replication (G1/G0) phase of the cell cycle [9] where the effects of DNA damage resulting from the IR would be less severe. The extended time period within this phase would allow the cell to undergo additional DNA repair. To study the effects of ON01210.Na on the cell cycle progression of HFL-1 cells, cells were treated with increasing concentrations of ON01210.Na for 24 hours and subjected to propidium iodide staining and flow cytometric analysis. HFL-1 cells treated with concentrations of ON01210.Na up to 50 µM showed a normal distribution of cells throughout the cell cycle, with a slight reduction in the number of cells in S-phase at 50 µM (Figure 2A). We extended the study by treating HFL-1 and human umbilical vein endothelial cells (HUVECs) with increasing concentrations and determined the number of viable cells after 96 hours of continuous exposure. The data from this dose response assay showed that continuous exposure to high concentrations (100 µM) of ON01210.Na did not result in cell death (Figure 2B). Although the growth of normal HFL-1 fibroblasts and normal endothelial cells (HUVECs) was reduced by 50% and 45%, respectively, with continuous exposure to 80–100 µM of ON01210.Na., cell viability was greater than 90% in both cell lines, even at the highest concentration of 100 µM (data not shown). The conclusion that ON01210 is not toxic to HFL-1 cells was further supported by our observation that treatment of HFL-1 cells with the compound at 100 µM concentration of the compound did not induce PARP cleavage or affect change in Annexin V staining (data not shown).

### Protection of Murine Hematopoietic Cells

**Figure 3:**
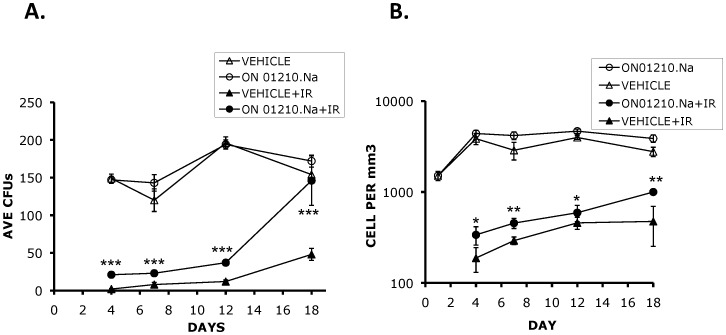
ON01210.Na protects murine pluripotent hematopoietic cells. (A) Colony forming assay of bone marrow cells derived from mice injected with ON01210.Na (500 mg/kg) at 24 hours and 15 minutes prior to whole body irradiation (5.5 Gy). Note the significant increase in colony numbers of cells isolated on day 18 post-IR from ON01210.Na+IR treated mice as compared with vehicle+IR (Control) (***P = <0.001). (B) White blood cell counts of ON01210.Na treated mice showed an increase recovery rate following exposure to IR. (*P = >0.05;**P = <0.05).

Acute radiation syndrome (ARS) is the result of IR damage to rapidly dividing cells of the bone marrow (BM), gastrointestinal tract (GI), skin and neurological tissues [3], [10]–[12]. Animal death due to exposure to lower radiation doses results from the ablation of the hematopoietic cell compartment, with death occurring between 12–24 days following exposure. To determine the ability of ON01210.Na to protect hematopoietic cells from radiation-induced death, mice were injected with the optimal dose of ON01210.Na (500 mg/kg) 24 hours and 15 minutes prior to radiation exposure at a sub-lethal dose of 5.5 Gy and the colony forming potential of their bone marrow as well as their white blood cell numbers were determined at various time points following radiation exposure. ON01210.Na treatment significantly increased the rate of recovery and differentiation of primitive bone marrow myeloid progenitor cells (Figure 3A). Vehicle treated mice exposed to 5.5 Gy of radiation had virtually no colony forming units on day 4 post-IR and in some experiments, there was complete inhibition of CFU formation beyond day 7. However, although there was a significant reduction of CFU numbers in mice that were treated with ON01210.Na in combination with radiation, the ON01210.Na-treated mice consistently retained a capability to form differentiated colonies. By 18 days, cells derived from ON01210.Na-treated mice recovered to the levels of non-irradiated cells, whereby the number of CFUs in the ON01210.Na-treated and non-irradiated samples were almost equivalent. On the other hand, the mice injected with the vehicle alone failed to recover, with the number of CFUs reaching only 30% of those from non-irradiated mice. This suggests that, unlike the irradiated control mice, ON01210.Na treated mice had a progenitor cell population that was never completely depleted by radiation exposure. Lineage specific protection was studied by identifying and tabulating the number of lineages, granulocyte (G), macrophage (M) and granulocyte/macrophage (GM) myeloid progenitors (data not shown). Recovery was observed in all three lineages with a slight bias towards the macrophage lineage, as we observed a slight increase in the CFU-M number when the mice were injected with ON01210.Na without radiation exposure. These results show that ON01210.Na was not only able to significantly increase the rate of recovery of bone marrow progenitor cells exposed to IR, but that it was also able to protect an existing fraction of cells. To determine the protection of peripheral white blood cells, whole blood was collected and the total number of white blood cells was determined at various times after radiation exposure. The results presented in Figure 3B show that the irradiated ON01210.Na treated mice harbor more white blood cells compared to the irradiated vehicle-treated controls, suggesting that ON01210.Na protected white blood cells from radiation exposure. In addition and consistent with the colony forming potential, the white blood cell numbers of mice treated with ON01210.Na recovered more rapidly by 18 days after IR exposure.

Our earlier studies have shown that when mice were irradiated with 8 Gy, the net survival was 88% for those treated with the drug 24 h and 15 min (two doses) before irradiation. Survival in the group given Ex-Rad only 15 min before irradiation was about 70% [7]. In order to correlate this survival data with protection of the bone marrow, we compared bone marrow protection of mice injected with a single dose of ON01210.Na at minus 15 minutes or minus 4 hours as well as with two doses at −24 hrs. and −15 min, which gave optimal protection. Even though a single injection just before radiation exposure was very effective in protecting bone marrow cells and inducing recovery of the bone marrow cell population, table 2 shows that the most efficient protection of the bone marrow was observed when the mice were injected at minus 24 hours and minus 15 minutes before radiation exposure. When ON01210.Na was injected as a single dose at 4 hours prior to radiation exposure, we observed protection of bone marrow cells, but the recovery rate was not significantly increased as compared to vehicle treated mice. These experiments imply that a single injection just before exposure will protect and increase the recovery of bone marrow cells after radiation exposure.

**Table 2:** Percent recovery of CFU based on schedule.

Day	TREATMENT SCHEDULE			
	Vehicle	−24 hr−15 min	−15 min	−4 hr
4	0%	14%	7%	5%
7	1%	16%	10%	7%
12	8%	25%	13%	9%
18	40%	100%	60%	40%

### *In Vitro* Protection of Human Bone Marrow

**Figure 4:**
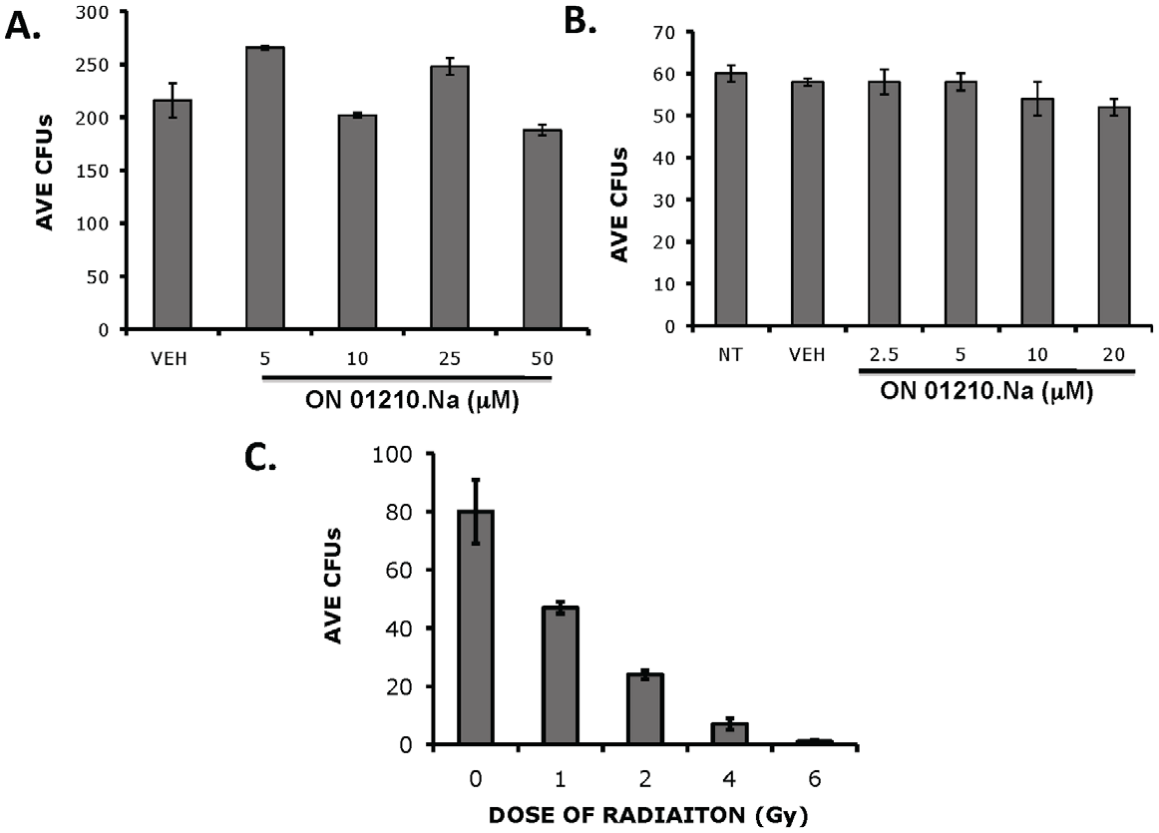
ON01210.Na treatment does not inhibit the colony forming potential of human bone marrow cells. Human bone marrow cells were treated with increasing concentrations of ON01210.Na for 2 hrs (A) or 24 hrs (B), followed by plating into methylcellulose. Total number of CFUs was determined and the average number (n = 3) was determined and plotted along with SEM. (C) Effect of ionizing radiation on the number of colony forming potential of human bone marrow cells.

To assess the ability of ON01210.Na to protect human bone marrow cells from radiation exposure, we developed a cytotoxicity assay using a standard colony forming assay as the end point. Human bone marrow cells were treated with various doses of ON01210.Na for 2 or 24 hours. The cells were then plated in methylcellulose and the total number of CFUs determined. As shown in Figure 4A and B, ON01210.Na did not inhibit the ability of human bone marrow to form colonies in methylcellulose at either timepoint. Treatment of cells for 24 hours with high doses of ON01210.Na (20 µM) also revealed no associated toxicity and the colony forming potential of treated cells showed no reduction compared to the vehicle treated controls (Figure 4B). These results suggest that ON01210.Na is non-toxic to human bone marrow cells at a concentration of 50 uM which was used to study the radio-protective effects of this compound.

**Figure 5:**
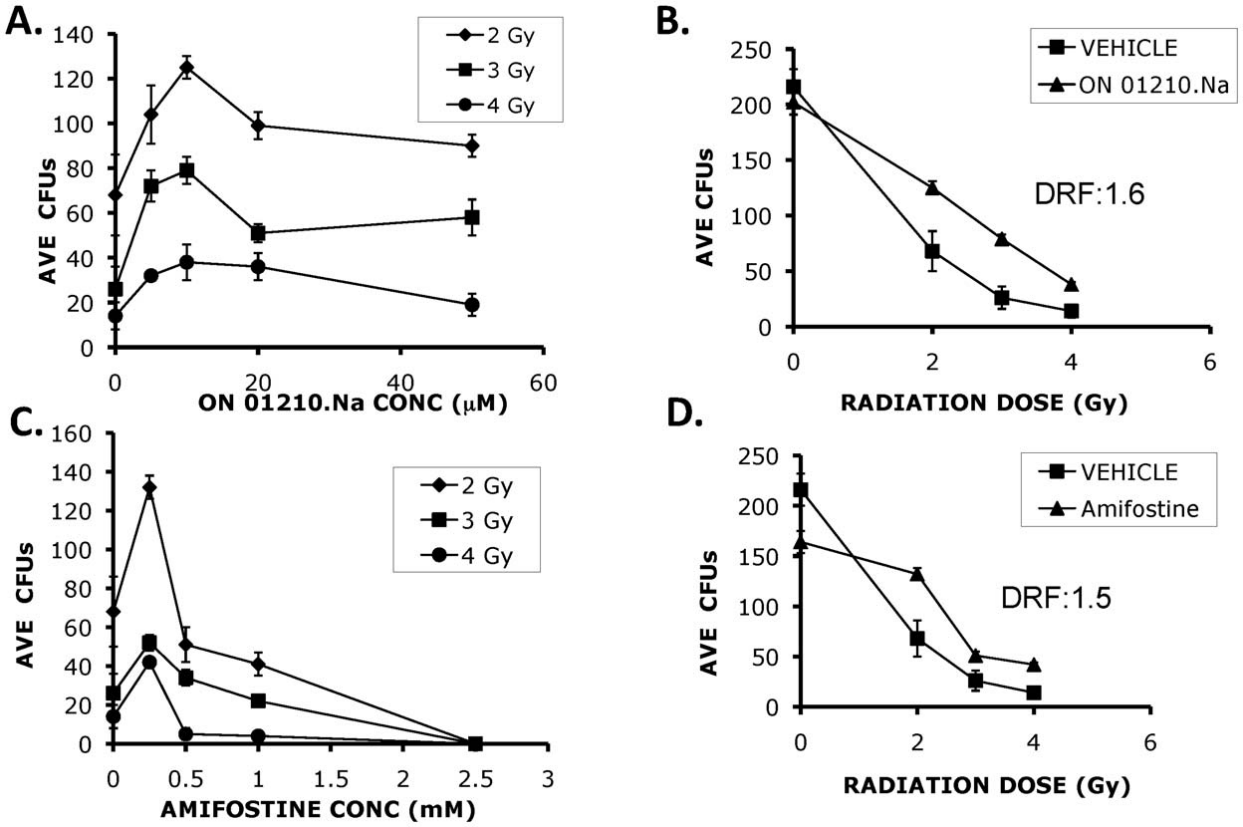
ON01210.Na protects human bone marrow cells from radiation exposure. Human bone marrow cells were pre-treated with varying concentrations of ON01210.Na (A) or Amifostine (C) and irradiated at 2, 3, and 4 Gy to identify the optimum dose of drug for radioprotection. Varying doses of radiation were used on human bone marrow cells that were pre-treated with 10 uM ON01210.Na (B) or 250 uM Amifostine (D) to calculate the dose reduction factor (DRF). ON01210.Na protected human bone marrow cells with a calculated DRF value of 1.6.

The next series of experiments were aimed at determining the radio-protective ability of ON01210.Na on human bone marrow cells in an *in vitro* radiation protection assay. In the first set of experiments, we generated a radiation dose response curve using bone marrow cells treated with vehicle and increasing doses of IR. As shown in Figure 4C, we observed the expected reduction in the surviving fraction of cells, whereby 76% to >90% of cells survived when exposed to 2****Gy–6****Gy of radiation. The cells were then pre-treated with increasing concentrations of ON01210.Na and then exposed to 2–4 Gy of IR. ON01210.Na provided dose dependent protection of human bone marrow cells at all three doses of IR. Concentrations of ON01210.Na above 10 µM did not increase protection from radiation (Figure 5A), suggesting that under these assay conditions, 10 µM was the optimal concentration for radiation protection. Plotting of a survival curve at the 10****µM concentration of ON01210.Na resulted in the calculation of a dose reduction factor (DRF) of 1.6 (Figure 5B). We also tested the radiation protection activity of amifostine, a free radical scavenger developed at the Walter Reed Army Institute of Research (WR-2721). Amifostine is a thiol that is broken down to its active metabolite in alkaline conditions to protect cells from radiation damage by acting as a free radical scavenger [13]. Concentrations were selected that closely mimicked therapeutic plasma concentrations [14], [15]. Amifostine protected human bone marrow cells in a narrow concentration range, with the optimal concentration being 250 uM (Figure 5C), which corresponds well with previously reported studies using mesenchymal and hematopoietic cells [15]. When the concentration of amifostine was increased to 500 uM, followed by radiation treatment, there was increased radiation sensitivity. Figure 5D shows the survival curve when human bone marrow cells were treated with 250 uM amifostine and at this concentration, the calculated DRF value for amifostine was 1.5. This data shows that ON01210.Na provides an equal level of protection when compared to amifostine, while exhibiting little or no toxicity. In addition, ON01210 was shown to be orally bioavailable which makes this compound ideal for radio-rotection [16].

### ON01210.Na Modulates the AKT Pathway to Promote Survival in Response to IR

**Figure 6:**
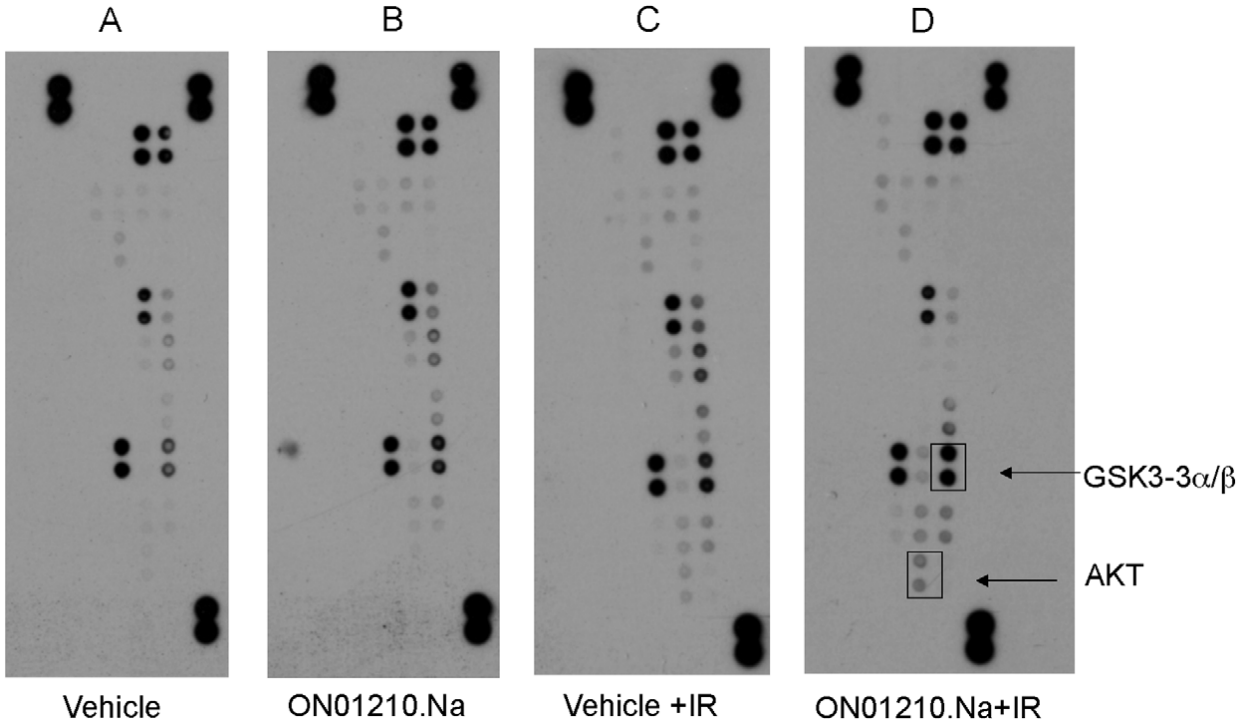
ON01210.Na treatment in combination with radiation alters the MAPK signaling pathway. Human Phospho-MAPK antibody arrays were treated with lysates from HFL-1 cells that were pre-treated with Vehicle (A), 20****uM ON01210.Na (B), Vehicle plus 10 Gy radiation (C), 20****uM ON01210.Na plus 10 Gy radiation (D). ON01210.Na treatment in combination with radiation increased the phosphorylation status of proteins associated with the AKT pathway.

In order to determine the mechanism of action of ON01210.Na, we employed an antibody array-based screening method. Because MAP kinases are known to play a critical role in cellular response to IR, we chose to determine the relative levels of phosphorylation of MAPKs and other serine/threonine kinases in cells exposed to radiation in the presence and absence of ON01210.Na. The membranes were incubated with lysates of HFL-1 cells that were subjected to radiation in the presence and absence of ON01210.Na. Treatment of HFLs with ON01210.Na in the absence of radiation did not affect the phosphorylation status of any of the proteins on the array compared to their vehicle-treated controls (Figure 6A–B). However, ON01210.Na was shown to activate the phosphorylation of AKT and GSK3α/β in HFL cells that were subjected to radiation treatment (Figure 6C–D), with an increase of over 250% and 450% in the phosphorylated forms of GSK3α/β and AKT, respectively compared to the vehicle-treated controls. Because AKT signaling is known to be an important mediator of cell survival [17]–[19] and GSK3ß inhibition has been directly correlated with cytoprotection from IR [20], these observations suggest that this pathway may be involved in ON01210.Na-mediated radioprotection.

### Time and Dose Dependent Activation of AKT Pathway

**Figure 7:**
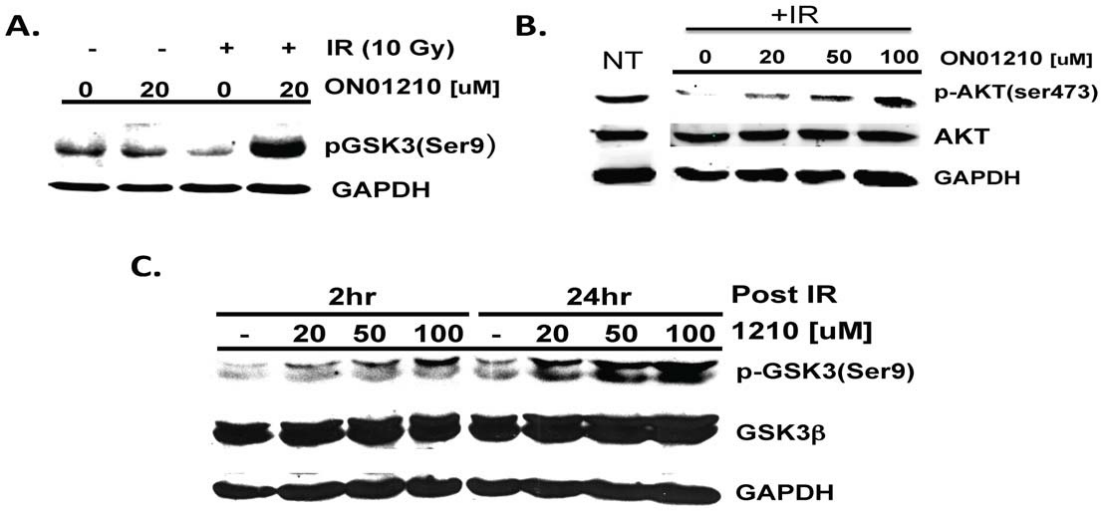
GSK3ß and AKT phosphorylation by ON01210.Na. (A) HFL-1 cells were treated with 20****uM ON01210.Na for 2 hours with or without exposure to 10 Gy of ionizing radiation. Cell lysates were subjected to western blot analysis using ßanti-phospho-GSK3ß-specific antibodies (B) HFL-1 cells were treated with increasing concentrations of ON01210.Na for 2 hours and irradiated with 10 Gy of ionizing radiation. Cell lysates were subjected to western blot analysis using anti-phospho-AKT (Ser473) specific and AKT antibodies. (C) HFL-1 cells were treated with increasing concentrations of ON01210.Na for 2 hours or 24 hrs and irradiated with 10 Gy ionizing radiation. Cell lysates were subjected to western blot analysis after 2 hours and 24 hours post radiation using anti-phospho-GSK3ß-specific and GSK3ß antibodies.

To confirm the results obtained from MAPK antibody array, we performed western blot analysis to determine the phosphorylation status of GSK3β and AKT (Figure 7). The activated, nonphosphorylated form of GSK3β has been shown to promote cell death under a number of cellular conditions such as neuronal degeneration and cranial irradiation (20), and is now a promising therapeutic target [21]. As shown in Figure 7A, treatment of cells with ON01210.Na alone does not result in any significant increase in the phosphorylation status of GSK3β. However, when the cells were treated with ON01210.Na for 2 hours followed by exposure to 10 Gy of IR, phosphorylation of GSK3β^Ser 9^ was found to increase in a time-dependent manner (Figure 7A and 7C). The observed increase in the GSK phosphorylation following radiation exposure was also found to be dose dependent at the two time points tested (2 Hr and 24 Hr). This increase in phosphorylation is expected to result in the inactivation of GSK3β. ON01210.Na treatment was also found to result in a dose-dependent increase the phosphorylation status of AKT, following exposure to 10****Gy of IR (Figure 7B), which is expected to result in its activation.

### ON01210.Na Modulates PI3-Kinase Activity

PI3-kinase is activated upon the stimulation of many survival/growth-associated pathways. Activation of PI3-kinase results in the production of 3′-phosphoinositol, leading to activation of AKT and phosphorylation of GSK3β and therefore constitutes an upstream step in AKT signaling pathway. The activation of PI3-Kinase pathway has been previously demonstrated to modulate a protective response to radiation [22], [23].

**Figure 8:**
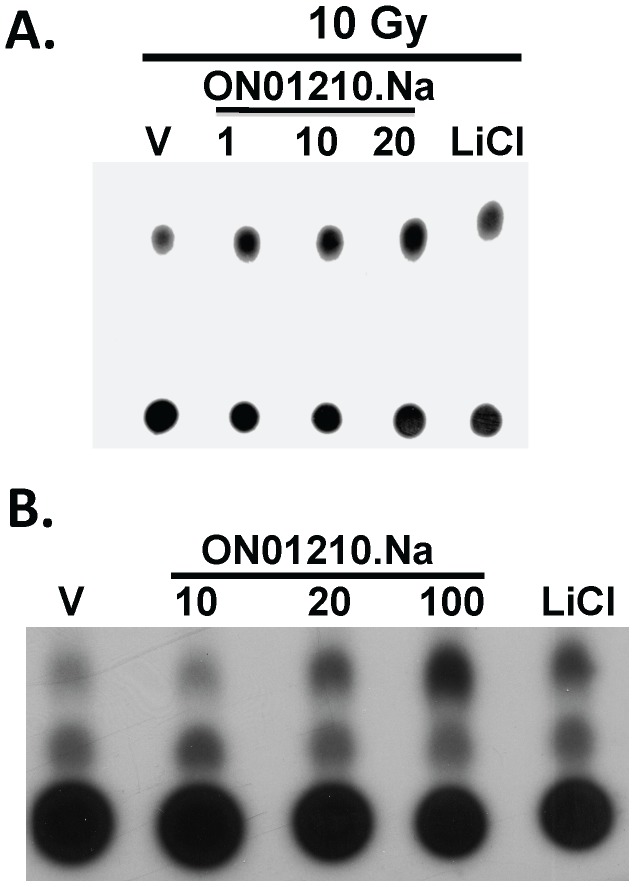
ON01210.Na increases PI3K activity in HFL-1 cells and murine bone marrow cells in response to radiation exposure. Lysates from HFL-1 cells (A) or mouse bone marrow cells (B) were treated with vehicle, increasing concentrations of ON01210.Na or 10****mM LiCl for 2 hours and then irradiated with 10 Gy ionizing radiation. Total protein lysates were immunoprecipitated with an anti-PI3 Kinase polyclonal antibody and IP- kinase assays were performed using L-α-Phosphatidylinositol as a substrate. The resulting phospholipids were resolved on TLC plates.

We therefore investigated the effect of ON01210.Na on the PI3-Kinase activity in HFL-1 cells subjected to radiation exposure. Although cells that were not exposed to radiation did not show a significant change in PI3-Kinase activity (data not shown), PI3-Kinase activity was increased when cells were treated with ON01210.Na and 10 Gy IR (Figure 8A). The level of PI3-Kinase activity was equal to or better than LiCl (10****mM), and previous studies have shown that treatment of cells with this compound results in the activation of PI3-kinase pathway and inhibition of GSK3 [24]. To further extend this data and correlate the mechanism with survival data, we conducted similar experiments using murine bone marrow cells. Murine bone marrow cells were treated with ON01210.Na for two hours prior to irradiation and their level of PI3-kinase activity was then determined. Similar to the fibroblast cell line, ON01210.Na activates the PI3-K in a dose dependent manner, with comparable levels of activation to that of 10****mM LiCl. This data strongly suggests that activation of this survival pathway is an important mechanism in ON01210.Na-mediated radiation protection.

## Discussion

In this communication, we describe the mechanism of action of ON01210.Na which acts as a potent radio-protective agent. ON01210.Na (Ex-RAD^®^) was identified as a radioprotectant, using a cell based screening technique, from a series of novel E-styryl benzones synthesized in our laboratory [5], [6]. This compound library contains agents that are potent inducers of cell death in tumor cells, and several of these compounds were found to modulate the activity of serine/threonine and tyrosine kinases in a selective manner [25], [26].

Acute Radiation Syndrome (ARS) results from DNA damage caused by radiation in rapidly dividing cells of the bone marrow (BM), gastrointestinal tract (GI), skin, and neurological tissue [3], [10]–[12]. The clinical manifestations of ARS are generally divided into 4 stages. The prodromal, early stage consisting of GI symptoms, such as diarrhea, nausea and vomiting can last for several days. This is followed by a full blown clinical illness period where symptoms arise due to depletion of the hematopoietic system, including circulating white and red blood cells, stem cells of both the bone marrow and of the GI tract. The last stage is either recovery or death depending on exposure levels. The hematopoietic syndrome develops at doses between 2–3 Gy, ranging from mild cytopenias at low doses to complete ablation of the bone marrow compartment at higher doses. Death is usually the result of severe immunosupression, leading to sepsis and other related clinical illnesses. There is an extensive body of literature on the effects of ionizing radiation, (i.e., gamma rays and neutrons) on the morbidity and mortality of various animal species. The mouse whole-body irradiation-survival model is accepted as an appropriate small animal species for studies in which several therapeutic parameters are being investigated and optimized [27]. Protection from radiation exposure has long been explored for use in both military and first responder scenarios. Protection against acute radiation syndrome (ARS) can be approached from three basic philosophies [27], [28] based on the time of treatment relative to radiation exposure and clinical signs and symptoms: [1] prophylaxis (pre-exposure), [2] mitigation (pre- to post-exposure, but before clinical symptoms), [3] treatment (post-exposure, after manifestation of clinical symptoms). An optimal radioprotectant would offer several ideal chemical and biological characteristics, including significant radiation protection/mitigation, low toxicity profile, high degree of bio-availability, protection of multiple organ targets (bone marrow, gastrointestinal tract and neurological), long shelf life, and compatibility with other drugs [12].

ON01210.Na (Ex-RAD^®^) was selected as our lead compound for radiation protection because it met many of the characteristics described above. Most importantly, this compound is soluble in water providing multiple formulation options for intravenous, subcutaneous and oral administration of the drug. In this report we show that ON01210.Na provides significant dose dependent radiation protection both *In Vitro* and *In Vivo* (Figure 1, Figure 5) by protecting the hematopoietic system. ON01210.Na, unlike many of the other compounds in this library, was non-toxic to normal cells derived from human fetal lung fibroblasts (HFL cells), HUVECs (Figure 2) and to normal human bone marrow cells (Figure 4). ON01210.Na has the chemical characteristics necessary for large-scale production and long-term stability necessary for administration to a large number of first responders (data not shown). This compound was also found to be water soluble and orally bio-available with very favorable pharmacokinetic properties in multiple routes of administration (data not shown).

In this report, we have examined the radioprotective effect of ON01210.Na on hematopoietic progenitor cells, bone marrow cells and whole blood which were collected from irradiated mice on consecutive intervals following radiation exposure. Based on the results from prior survival studies (100% protection), initial studies were carried out by injecting mice with 500 mg/kg of ON01210.Na suspension subcutaneously at 24 hours and 15 minutes before exposure to 5.5 Gy of radiation. Bone marrow samples were harvested on day 4, 7, 12 and 18 days after radiation exposure, and the total number of colony forming units (CFU) was enumerated. Our results show that ON01210.Na treatment significantly increased the rate of recovery and differentiation of primitive bone marrow progenitor cells (Figure 3, Table 2). By 18 days, ON01210.Na treated mice had completely normal levels of cells capable of forming differentiated colonies. Under identical conditions, the mice injected with the vehicle alone did not recover well, with the number of CFUs reaching only 30% of those from non-irradiated mice. Vehicle treated mice exposed to 5.5 Gy of radiation had virtually no colony forming units on day 4 post-radiation exposure and in some experiments, there was complete inhibition of CFU formation beyond day 7. However, although there was a significant reduction of CFU numbers in mice that were treated with ON01210.Na plus radiation, the ON01210.Na treated mice consistently retained a capability to form differentiated colonies. This suggests that unlike the vehicle irradiated control mice (treated with vehicle only), ON01210.Na treated mice had a progenitor cell population that was not completely depleted by radiation exposure. Lineage specific protection was studied by identifying and tabulating the progenitors along the myeloid lineage; granulocytes (CFU-G), macrophages (CFU-M) and granulocyte-macrophage (CFU-GM), with a slight bias towards the macrophage lineage. There was also a slight increase in the macrophage colony number when the mice were injected with ON01210.Na without radiation exposure.

In order to determine the optimal schedule of ON01210.Natreatment for maximum protection of the bone marrow, we compared bone marrow protection of mice injected with a single dose of ON01210.Na at minus 15 minutes or minus 4 hours with mice injected with the optimal dose (minus 24 hours and minus 15 minutes. While the most efficient protection of the bone marrow was found when the mice were injected at minus 24 hours and minus 15 minutes before radiation exposure (Table 2), a single injection just before exposure was also very effective in protecting bone marrow cells and inducing recovery of the bone marrow cells following radiation exposure. When ON01210.Na was injected as a single dose at 4 hours before radiation exposure, we observed protection of bone marrow cells, but the recovery rate was not significantly increased. These experiments imply that a single injection just before exposure will protect and increase the recovery of bone marrow cells after radiation exposure. This correlates well with the survival studies which showed that a single injection of ON01210.Na 15 minutes prior to radiation exposure provided 100% protection.

Because it is not possible to test ability of ON01210.Na to protect the human hematopoietic system from radiation exposure the *in vivo*, we attempted to mimic this scenario by using an *in vitro* system that employed normal donor bone marrow samples. These studies involved plating of freshly harvested human donor bone marrow samples following radiation exposure with and without ON01210.Na treatment. The studies also compared the radiation protection activity of ON01210.Na to that of amifostine, a free radical scavenger developed at the Walter Reed Institute in Washington DC (WR-2721). Cytotoxicity testing of human bone marrow cells revealed that ON01210.Na did not inhibit the growth and differentiation of normal human bone marrow cells. The 2 hr data (Figure 4A) clearly showed that short-term exposure to ON01210.Na is non-toxic up to a concentration of 50 µM. Continuous treatment of cells for 24 hours with high doses of ON01210.Na (20 µM) did not reveal any appreciable toxicity (Fig. 4B) and analysis of the colony forming potential of these cells showed that ON01210.Na treated cells differentiated with equal potential as the vehicle treated controls. Under similar conditions, a 3 hr. exposure to amifostine was toxic to human bone marrow cells at concentrations above 2.5****mM (data not shown), and pretreatment of human bone marrow cells prior to radiation increased the sensitivity to ionizing radiation if the concentration was above 0.5 mM (Figure 5C). A 2-fold increase of amifostine also resulted in a negative impact on survival. ON01210.Na had a much wider window of efficacy, and even a 10-fold increase in ON01210.Na concentration in combination with IR resulted in a survival benefit (Figure 5A). When we plotted the survival curves at the optimal concentration for both ON01210.Na and amifostine (Fig. 5B, 6D), ON01210.Na had a DRF value of 1.6 while amifostine had a DRF value of 1.5. These data indicate that ON01210.Na was as effective at protecting human bone marrow cells from radiation exposure as amifostine, and exhibited a superior safety profile at a wide range of concentrations.

Our studies also suggest that ON01210.Na offers a novel mechanism for radiation protection involving intracellular signal transduction, damage sensing, and DNA repair pathways. Our earlier studies showed that ON01210.Na treatment reduces the amount of DNA damage (or enhances DNA repair) caused by irradiation of cells, as assessed by the “Comet” assay, enabling more rapid recovery of cells [7]. We have investigated effects on several cellular damage sensing and signaling pathways important for response and repair following a cell’s exposure to radiation. These studies revealed that treatment of cells with ON01210.Na prior to radiation exposure does not adversely affect the early cellular responses to radiation exposure, but influences later signaling events that contribute to reduction in cellular apoptosis caused by the signaling cascade activated upon exposure to radiation [7].

In addition to the observed effects of ON01210.Na on the p53 signaling pathway [7], this report shows that ON01210.Na activates the PI3K/AKT signaling cascade, which is known to be an important cell survival pathway. Akt, also known as protein kinase B, plays a central role in the response to many extracellular and receptor activated signaling cascades that often lead to cell survival [29]. This anti-apoptotic response is generated through the activation of PI3-kinase, resulting in the production of 3′-phosphorylated phosphoinositides causing a recruitment of AKT to the membrane where it is activated through phosphorylation events. One important downstream target of AKT is glycogen synthase kinase 3ß (GSK3ß). The activated, non-phosphorylated, form of GSK3ß has been shown to promote cell death under a number of cellular conditions such as neuronal degeneration and cranial irradiation [20], and is now a promising therapeutic target [21]. GSK3ß also plays a role as a key regulator during hematopoietic stem cell repopulation [30] through regulation of several key pathways including Wnt [31] Hedgehog [32] and Notch [33]. It has recently been shown that GSK3ß inhibitors act directly on primitive cells (Lin^−^c-Kit^+^Sca1^−^ cells) by increasing their proliferation rate leading to enhanced CFU output [370. These studies suggest that *in vivo* administration of GSKß inhibitors would enhance long-term function of hematopoietic stem cells. It is therefore not surprising that the same outcome results from ON01210.Na treatment of cells subjected to irradiation. In addition, recent studies show that AKT regulates the level of reactive oxygen species (ROS) to maintain the function of the long-term hematopoietic stem cells [34], which could further enhance the bone marrow protective properties of ON01210.Na.

We have previously reported that treatment of cells with ON01210.Na followed by radiation results in the down-regulation of the steady state levels of p53, p21, Bax and c-abl proteins [7]. The combination of p53 down-regulation (pro-apoptotic) and up-regulation of the Akt pathway (pro-survival) mediated by ON01210.Na presumably leads to increased survival and repopulation of the hematopoietic and endothelial cells [35,36). ON01210.Na has completed a phase I clinical trial of normal human subjects to evaluate the safety, tolerability and pharmacokinetic profiles of ON01210.Na These studies have confirmed the safety profile of ON01210.Na in human subjects (data not shown). ON01210.Na formulated as a solution is now being tested for post radiation mitigation as well as for oral dosing for both pre- and post-radiation exposure scenarios. These clinical studies are likely to reveal the best way to utilize ON01210.Na to protect human subjects from radiation injury.

## Materials and Methods

### Compounds

ON01210.Na ((E)-4-carboxystyryl-4-chlorobenzylsulfone, sodium salt) (C_16_H_12_ClNaO_4_S, molecular weight of 358.79) is under development by Onconova Therapeutics, Inc. (Newtown, NJ) as a radioprotectant. The stock free acid (ON01210) was synthesized by Chem Pacific (Batch # CHP-0219021) and was used to synthesize the sodium salt (ON01210.Na batch #ON062604-1210.Na). The free acid was dissolved in DMSO and the ON01210.Na was prepared immediately before each experiment by dissolving in sterile ddH_2_O (Millipore). Amifostine, clinical sample 17315-7253-3, (Alza/US Bioscience) was a gift from Dr. Alan Alfieri, Albert Einstein Medical Center, NY.

### Cell Culture

HFL-1 cells were purchased from ATCC and grown in DMEM supplemented with 10% FBS (Cellgeneration, CO) and Pen-Strep solution and tested following two passages in culture. HUVECs were purchased from Cambrex/Clonetics and cultured in endothelial growth medium according to instructions provided by the supplier. Compound screening was performed by plating 3000 HFL-1 cells per well per ml in 24 well dishes. The cells were then treated with various concentrations of each compound (2.5–10 uM) for 24 hours. The plates were then irradiated with 10 Gy, cells were washed and plates were incubated for 96 hours. After the 96 hours, each well was trypsinized and replated into 100 mm^2^ dishes 96 hours later. Plates were stained and colonies were enumerated 3 weeks later. For cell counts, the cells were plated into 6 well dishes at a cell density of 1.0×10^5^ cells/ml/well and ON01210.Na was added 24 hours later at various concentrations. Cell counts were determined after 96 hours of treatment. The total number of viable cells was determined using trypan blue exclusion.

### Flow Cytometric Analysis

HFL-1 cells (1×10^6^ cells per 100****mm^2^) were treated with various concentrations of ON01210 (1.0–50 µM) or an equal volume of DMSO. The cells were harvested at 24 hrs post-treatment, washed phosphate buffered saline, and subsequently fixed in ice cold 70% ethanol. The fixed samples were then prepared for FACS analysis by first washing the ethanol treated cells in PBS, followed by staining in a solution containing 100 ug/ml propidium iodide/ 0.5 mg/ml RNase A at 37°C. The data for flow cytometry were acquired on a FACScan, (BD Biosciences, San Jose, CA. USA), equipped with a 488****nm Argon laser which excites PI. At least 10 thousand cells were collected. The cell cycle profiles were analyzed and calculated by using the CELLQUEST / MODIFIT LT Software (Verity Soft ware House, Topsham, ME).

### Murine Hematopoietic Radiation Protection Studies

ON01210.Na formulated in a potassium-phosphate buffer (pH 7.6) was injected into Female C3H/HEJ mice at 7 weeks of age with an average weight of 25 grams at a dose of 500 mg/kg (12.5 mg (0.25 ml) of ON01210.Na as a suspension). The mice were injected subcutaneously with freshly prepared ON01210.Na or equal volume of vehicle into the back between the shoulder blades using a 23 or 27-1/2 gauge needle, respectively.

For the irradiation studies, mice were irradiated at 5.5 Gy for 3.67 minutes at 150.8 rads/min or 149.0 rads/min, study 1 and study 2, respectively, using a Shepherd Mark I irradiator with a 137-Cesium source using a glass container while rotating at position 2. Groups of 5–6 mice were irradiated at a time with no anesthesia. At the indicated times, mice were euthanized and whole blood was collected using cardiac puncture and collected into heparinized tubes for white blood cell counts. An aliquot (0.2 ml) of blood was added to 15 ml centrifuge tubes containing 10 ml of ammonium chloride (StemCell Technolgies, Vancouver, British Columbia) to lyse the red blood cells. White blood cell counts performed by combining 10****µl of cell suspension with 90****µl of methylene blue/acidic acid solution and the number of white blood cells counted using using a hemacytometer.

For studies using murine bone marrow, fresh bone marrow was isolated from the femurs and tibias of mice and 1.0×10^5^ cells were plated in a 60****mm dish containing MethoCult™ GF M3534 (StemCell Technologies, Vancouver, British Columbia, Cat #03534, for CFU-GM only), which has been formulated to support optimal growth of mouse granulocyte and macrophage progenitors. Statistical analysis was performed using two array Students t-test.

### *In Vitro* Protection of Human Bone Marrow Cells

Human bone marrow was obtained from a commercial source (Lonza, MD) and collected from healthy human volunteers after signed consent following all IRB guidelines. Three independent samples of human bone marrow cells were used for three series of studies. Cells were processed for assay by first lysing contaminating red blood cells. The remaining bone marrow cells were counted and treated (1.0×10^5^) with vehicle or increasing concentrations of test material (ON01210.Na or amifostine) for various periods of time. Following treatments, cells were cultured directly (cytotoxicity testing), or exposed to varying doses of IR before being cultured (radiation protection testing). For cytotoxicity assays, cells (1.0×10^5^ cells/ml HPGM) were treated with various concentrations of ON01210.Na or vehicle without radiation treatment for 2 or 24 hours. The cells were washed and plated into methocult (StemCell Technologies, Vancouver, British Columbia, Cat #H4434, for CFU-GM expansion) using gridded dishes. The total number of colony forming units (CFUs) was determined 14 days post-plating by microscopic observation using an Olympus IMT-2 microscope. For the radiation protection assay, the purified human bone marrow cells (1.0×10^5^ cells/ml were treated with various concentrations of ON01210.Na or Amifostine for 2–3 hours at 37°C in HPGM (Lonza, MD cat# PT-3926). The cells were then irradiated at 2****Gy, 3****Gy or 4****Gy using a Shepherd Mark I irradiator with a ^137^Cesium source at a dose rate of 265.17 rads/min, returned to the incubator for 1 hour and then plated onto methocult. After a 14-day incubation period, the number of colony forming units was determined.

### Antibody Array-based Screening

Exponentially growing HFL-1 cells were treated with 20 µM ON01210.Na for 2 hours and exposed to 10****Gy IR with ^137^Cs. After a 4 hour period, the cells were harvested, washed in PBS and solubilized at 1×10^7^ cells/ml in the lysis buffer supplied by the manufacturer (R&D Systems Inc., Minneapolis, MN, USA). The cells were lysed at 4°C for 30 minutes, clarified and 200****ug of clarified protein lysate in a volume of 250****µl was used for the Proteome Profiler™ Array Human Phospho-MAPK Array Cat #: ARY002) according to the protocol supplied by the manufacturer.

### Western Blotting

Exponentially growing HFL-1 cells were treated with as indicated and exposed to 10****Gy IR. These cells were harvested, washed in PBS and lysed using RIPA buffer (1% NP40 NP 40, 0.5% Na-Deoxycholate, 50****mM Sodium Fluoride, 0.1% SDS, 2****mM EDTA, 1****mM PMSF, 2****µg/ml Leupeptin, 4****µg/ml aprotinin, 1****mM Benzamidine, 200****mM Na_3_VO_4_, 0.1% ß-mercaptoethanol). The samples were resolved on an 10% SDS-acrylamide gel, transferred to nitrocellulose and subjected to western blot analysis. All membranes were blocked with blocking solution (LI-COR^®^, Odyssey Infrared Imaging System #927-40000) and probed overnight with primary antibodies as indicated. The membranes then washed , probed with the appropriate labeled secondary antibodies (LI-COR, Lincoln, NB) and washed again prior to analysis using a LI-COR^®^ Odyssey^®^ Infrared Imaging System. The following antibodies were used for the immunoblotting experiments: AKT (Cell Signaling Technology #9272), phospho-AKT (Ser473) (Cell Signaling Technology #9271), GSK-3ß (1F7) (Santa Cruz Biotechnology Inc. SC-S3931), and phspho-GSK-3ß(Ser9) (Cell Signaling Technology #9336).

### PI3-Kinase Assay

PI3-kinase assays were performed using exponentially growing HFL-1 or freshly harvested murine bone marrow cells that were treated with increasing concentrations of ON01210.Na for 2 hours and then irradiated with 10 Gy IR. These cells were then returned to the incubator for 2 to 24 hours and lysed in HEPES pH 7.5 lysis buffer. PI-3K was immunoprecipitated using an anti-PI3 Kinas polyclonal antibody (Cell Signaling Technology #4249) for 2 hours at 4°C. Protein A/G PLUS-Agarose (Santa Cruz Biotechnologies SC-2003) was incubated with immunoprecipitates for 8–16 hours at 4°C and the resulting immunoprecipitates washed with twice HEPES pH 7.5 lysis buffer and once with the kinase buffer (20****mM Tris pH 7.5, 1****mM EGTA, 10****mM MgCl2, 2****mM DTT, 0.01% NP-40). L-α-Phosphatidylinositol (12.5****mM) and ATP (10****µM) were added to the kinase buffer (60****µl per sample) and incubated at 30°C for 30 minutes. The reaction was stopped by addition of 100****µl of 1****N HCl and extracted by addition of 200****µl CHCl3/CH3OH (1∶1). The extracted samples were vortexed, centrifuged and the lower organic phases containing phospholipids were dried at 27°C for 2 hours. The dried samples were resuspended in 10****µl of PI-4-P standard (0.5****ml CHCl3, 0.5****ml CH3OH, 2.5****µl HCl) and spotted on TLC plates (VWR). The spotted plate was subjected to thin layer chromatography in CHCl3/CH3OH/NH4OH (40∶40∶15). The TLC plate was dried and subjected to autoradiography.

## References

[1] Paris F, Grassme H, Cremesti A, Zager J, Fong Y, et al (2001) Natural ceramide reverses Fas resistance of acid sphingomyelinase(−/−) hepatocytes. J Biol Chem 276: 8297–8305. 11096096 10.1074/jbc.M008732200

[2] Langell J, Jennings R, Clark J, Jr WJ (2008) Pharmacological agents for the prevention and treatment of toxic radiation exposure in spaceflight. Aviat Space Environ Med 79: 651–660. 18619123 10.3357/asem.2113.2008

[3] Waselenko JK, MacVittie TJ, Blakely WF, Pesik N, Wiley AL, et al (2004) Medical management of the acute radiation syndrome: recommendations of the Strategic National Stockpile Radiation Working Group. Ann Intern Med 140: 1037–1051. 15197022 10.7326/0003-4819-140-12-200406150-00015

[4] Dumont F Le Roux A (2010) Bischoff (2010) Radiation countermesure agents: an update. Expert Opin. Ther. Patents 20(1): 73–101. 10.1517/13543770903490429 20021286

[5] Reddy EP, Reddy MVR (2004) “(E)-Styryl Sulfone Anticancer Agents”. In: USPTO, editor. U.S.: Temple University.

[6] Reddy EP, Reddy MVR, Cosenza SC (2002) Method for protecting cells and tissues from ionizing radiation toxicity with alpha, beta unsaturated aryl sulfones. Temple University, Onconova Therapeutics, Inc.

[7] Ghosh SP, Perkins MW, Hieber K, Kulkarni S, Kao TC, et al (2009) Radiation protection by a new chemical entity, Ex-Rad: efficacy and mechanisms. Radiat Res 171: 173–179. 19267542 10.1667/RR1367.1

[8] Hall EJ, Giaccia AJ (2006) Radiobiology for the radiologist. Philadelphia: Lippincott Williams & Wilkins. ix, 546 p.

[9] Denekamp J (1986) Cell kinetics and radiation biology. Int J Radiat Biol Relat Stud Phys Chem Med 49: 357–380. 3510997 10.1080/09553008514552591

[10] Dainiak N (2002) Hematologic consequences of exposure to ionizing radiation. Exp Hematol 30: 513–528. 12063018 10.1016/s0301-472x(02)00802-0

[11] Dainiak N, Waselenko JK, Armitage JO, MacVittie TJ, Farese AM (2003) The hematologist and radiation casualties. Hematology Am Soc Hematol Educ Program: 473–496. 10.1182/asheducation-2003.1.473 14633795

[12] Hosseinimehr SJ (2007) Trends in the development of radioprotective agents. Drug Discov Today 12: 794–805. 17933679 10.1016/j.drudis.2007.07.017

[13] Porta C, Maiolo A, Tua A, Grignani G (2000) Amifostine, a reactive oxigen species scavenger with radiation- and chemo-protective properties, inhibits in vitro platelet activation induced by ADP, collagen or PAF. Haematologica 85: 820–825. 10942928

[14] List AF, Heaton R, Glinsmann-Gibson B, Capizzi RL (1998) Amifostine stimulates formation of multipotent and erythroid bone marrow progenitors. Leukemia 12: 1596–1602. 9766505 10.1038/sj.leu.2401151

[15] Ramdas J, Warrier RP, Scher C, Larussa V (2003) Effects of amifostine on clonogenic mesenchymal progenitors and hematopoietic progenitors exposed to radiation. J Pediatr Hematol Oncol 25: 19–26. 12544769 10.1097/00043426-200301000-00006

[16] Suman S, Datta K, Doiron K, Ren C, Kumar R, et al (2012) Radioprotective effects of ON01210.Na upon oral administration. J Radiat Res. 53: 368–76. 10.1269/jrr.11191 22739006

[17] Datta SR, Brunet A, Greenberg ME (1999) Cellular survival: a play in three Akts. Genes Dev 13: 2905–2927. 10579998 10.1101/gad.13.22.2905

[18] Franke TF (2008) PI3K/Akt: getting it right matters. Oncogene 27: 6473–6488. 18955974 10.1038/onc.2008.313

[19] Bonnaud S, Niaudet C, Legoux F, Corre I, Delpon G, et al (2010) Sphingosine-1-phosphate activates the AKT pathway to protect small intestines from radiation-induced endothelial apoptosis. Cancer Res 70: 9905–9915. 21118968 10.1158/0008-5472.CAN-10-2043

[20] Thotala DK, Hallahan DE, Yazlovitskaya EM (2008) Inhibition of glycogen synthase kinase 3 beta attenuates neurocognitive dysfunction resulting from cranial irradiation. Cancer Res 68: 5859–5868. 18632640 10.1158/0008-5472.CAN-07-6327

[21] Eldar-Finkelman H (2002) Glycogen synthase kinase 3: an emerging therapeutic target. Trends Mol Med 8: 126–132. 11879773 10.1016/s1471-4914(01)02266-3

[22] Gupta AK, Bakanauskas VJ, McKenna WG, Bernhard EJ, Muschel RJ (2001) Ras regulation of radioresistance in cell culture. Methods Enzymol 333: 284–290. 11400344 10.1016/s0076-6879(01)33063-x

[23] Gupta AK, Cerniglia GJ, Mick R, Ahmed MS, Bakanauskas VJ, et al (2003) Radiation sensitization of human cancer cells in vivo by inhibiting the activity of PI3K using LY294002. Int J Radiat Oncol Biol Phys 56: 846–853. 12788194 10.1016/s0360-3016(03)00214-1

[24] Yazlovitskaya EM, Edwards E, Thotala D, Fu A, Osusky KL, et al.. (2006) Lithium treatment prevents neurocognitive deficit resulting from cranial. 10.1158/0008-5472.CAN-06-2740 17145862

[25] Gumireddy K, Baker SJ, Cosenza SC, John P, Kang AD, et al (2005) A non-ATP-competitive inhibitor of BCR-ABL overrides imatinib resistance. Proc Natl Acad Sci U S A 102: 1992–1997. 15677719 10.1073/pnas.0408283102 PMC546016

[26] Reddy MV, Mallireddigari MR, Cosenza SC, Pallela VR, Iqbal NM, et al (2008) Design, synthesis, and biological evaluation of (E)-styrylbenzylsulfones as novel anticancer agents. J Med Chem 51: 86–100. 18088089 10.1021/jm701077b

[27] Stone HB, Moulder JE, Coleman CN, Ang KK, Anscher MS, et al (2004) Models for evaluating agents intended for the prophylaxis, mitigation and treatment of radiation injuries. Report of an NCI Workshop, December 3–4, 2003. Radiat Res 162: 711–728. 15548121 10.1667/rr3276

[28] Moulder JE, Cohen EP (2007) Future strategies for mitigation and treatment of chronic radiation-induced normal tissue injury. Semin Radiat Oncol 17: 141–148. 17395044 10.1016/j.semradonc.2006.11.010

[29] Song G, Ouyang G, Bao S (2005) The activation of Akt/PKB signaling pathway and cell survival. J Cell Mol Med 9: 59–71. 15784165 10.1111/j.1582-4934.2005.tb00337.x PMC6741304

[30] Trowbridge JJ, Xenocostas A, Moon RT, Bhatia M (2006) Glycogen synthase kinase-3 is an in vivo regulator of hematopoietic stem cell repopulation. Nat Med 12: 89–98. 16341242 10.1038/nm1339

[31] Yost C, Torres M, Miller JR, Huang E, Kimelman D, et al (1996) The axis-inducing activity, stability, and subcellular distribution of beta-catenin is regulated in Xenopus embryos by glycogen synthase kinase 3. Genes Dev 10: 1443–1454. 8666229 10.1101/gad.10.12.1443

[32] Jia J, Tong C, Jiang J (2003) Smoothened transduces Hedgehog signal by physically interacting with Costal2/Fused complex through its C-terminal tail. Genes Dev 17: 2709–2720. 14597665 10.1101/gad.1136603 PMC280620

[33] Foltz DR, Santiago MC, Berechid BE, Nye JS (2002) Glycogen synthase kinase-3beta modulates notch signaling and stability. Curr Biol 12: 1006–1011. 12123574 10.1016/s0960-9822(02)00888-6

[34] Juntilla MM, Patil VD, Calamito M, Joshi RP, Birnbaum MJ, et al (2010) AKT1 and AKT2 maintain hematopoietic stem cell function by regulating reactive oxygen species. Blood 115: 4030–4038. 20354168 10.1182/blood-2009-09-241000 PMC2875090

[35] Paris F, Fuks Z, Kang A, Capodieci P, Juan G, et al (2001) Endothelial apoptosis as the primary lesion initiating intestinal radiation damage in mice. Science 293: 293–297. 11452123 10.1126/science.1060191

[36] Bonnaud S, Niaudet C, Legoux F, Corre I, Delpon G, et al (2010) Sphingosine-1-phosphate activates the AKT pathway to protect small intestines from radiation-induced endothelial apoptosis. Cancer Res 70: 9905–9915. 21118968 10.1158/0008-5472.CAN-10-2043

